# Regulation of FSH Synthesis by Differentially Expressed miR-488 in Anterior Adenohypophyseal Cells

**DOI:** 10.3390/ani11113262

**Published:** 2021-11-15

**Authors:** Hao-Qi Wang, Wen-Hua Wang, Cheng-Zhen Chen, Hai-Xiang Guo, Hao Jiang, Bao Yuan, Jia-Bao Zhang

**Affiliations:** Department of Laboratory Animals, College of Animal Sciences, Jilin University, Changchun 130062, China; hqwang1997@163.com (H.-Q.W.); wangwenhua1005@yeah.net (W.-H.W.); chencz@jlu.edu.cn (C.-Z.C.); guohx20@mails.jlu.edu.cn (H.-X.G.); jhhaojiang@jlu.edu.cn (H.J.)

**Keywords:** pituitary, follicle-stimulating hormone, gonadotropin-releasing hormone, microRNA, animal reproduction

## Abstract

**Simple Summary:**

GnRH and FSH play an important regulatory role in the reproductive activities of mammals. At present, many artificially synthesized GnRH analogues have been used in the regulation of cattle reproduction and the clinical treatment of various reproductive diseases. This study explored the potential mechanism of miR-488 in GnRH regulation of FSH synthesis and secretion and provides a theoretical basis for the application of GnRH analogue in cattle artificial breeding. We hope to provide a research foundation for improving the processing procedures of cattle estrus control and the domestic application of hormone products.

**Abstract:**

Gonadotropin-releasing hormone (GnRH), which is synthesized and released by the hypothalamus, promotes the synthesis and secretion of follicle-stimulating hormone (FSH), thereby regulating the growth and reproduction of animals. GnRH analogues have been widely used in livestock production. MiRNAs, which are endogenous non-coding RNAs, have been found to play important roles in hormone regulation and other physiological processes in recent years. However, the roles of miRNAs in GnRH-mediated regulation of FSH secretion have rarely been studied. Herein, we treated bovine anterior adenohypophyseal cells with an exogenous GnRH analogue and found that miR-488 was differentially expressed. Through a combination of TargetScan prediction and dual luciferase reporter analysis, miR-488 was confirmed to be able to target the FSHB gene. Based on this finding, we verified the expression of Fshβ and Lhβ mRNA in the rat adenohypophysis before and after exogenous GnRH treatment in vivo and in vitro. Experiments on rat anterior adenohypophyseal cells showed that overexpression of miR-488 significantly inhibited Fshβ expression and FSH synthesis, while knockdown of miR-488 had the opposite effects. Our results demonstrate that GnRH relies on miR-488 to regulate FSH synthesis, providing additional useful evidence for the significance of miRNAs in the regulation of animal reproduction.

## 1. Introduction

Reproductive hormones are involved in every stage of mammalian reproduction. The reproductive endocrine system, which is composed of the hypothalamic–pituitary–gonadal (HPG) axis, finely regulates reproductive processes in mammals [1]. The hypothalamus-secreted gonadotropin-releasing hormone (GnRH) regulates the synthesis and secretion of gonadotropins in the adenohypophysis, thereby regulating gonadal function [2]. Follicle-stimulating hormone (FSH), a gonadotropin secreted by the adenohypophysis, plays a vital role in the regulation of gametogenesis and maturation [3,4,5,6]. At present, reproductive hormones, such as GnRH and FSH, are widely used to regulate animal reproduction [7,8]. Combined treatment with GnRH and prostaglandin is a commonly used technique for estrus synchronization in cattle. GnRH induces follicle development and promotes ovulation by promoting the release of FSH and luteinizing hormone (LH), thereby increasing the conception rate in cows. Applying gonadorelin, a type of synthetic GnRH, for estrus synchronization can greatly reduce the cost of hormone use for animal production. However, the lack of understanding of the HPG axis regulation mechanism has restricted the application of reproductive hormones for animal production pursuits.

MicroRNAs (miRNAs) are small non-coding RNAs encoded by the genome. MiRNAs can inhibit transcription and degrade mRNA by recognizing the 3’UTR sequences of target mRNA molecules and acting as post-transcriptional regulators of gene expression [9]. It has been reported that miRNAs are involved in cell proliferation [10], cell differentiation [11], apoptosis [12], cell adhesion [13], cell migration [14], hematopoiesis [15], neurogenesis [16], and many other basic biological processes. Some research has also yielded insights into miRNA-mediated regulation of reproductive hormone synthesis and secretion. For example, miR-21-3p and miR-433 can participate in the regulation of FSH synthesis and secretion by targeting *Fshβ* [17]. However, there have been few studies on the roles of miRNAs in the processes by which GnRH regulates the expression of *Fshβ* and the synthesis and secretion of FSH.

In this study, we treated 30 cows with a combination of gonadorelin and prostaglandin and discovered that gonadorelin could mimic GnRH to regulate FSH synthesis and secretion. In addition, we found that the miRNA miR-488 was differentially expressed in the pituitary gland before versus after exogenous GnRH treatment and verified the regulatory effects of miR-488 on *Fshβ* expression and FSH synthesis in rat anterior adenohypophyseal cells in vitro.

## 2. Materials and Methods

### 2.1. Animals and Ethics

The research was carried out with the approval of the College of Animal Science of Jilin University and the Institutional Animal Care and Use Committee of Jilin University (201705026). Healthy eight-week-old male Sprague–Dawley (SD) rats were kept in an enclosed laboratory animal facility for experimental research under a 12/12 h light/dark cycle with free access to food and water. In addition, thirty cows in diestrus with no dominant follicles (as determined by rectal examination) were selected for follow-up experiments. All research procedures were completed in strict accordance with animal welfare laws and regulations and with animal welfare ethics.

### 2.2. Tissue Collection

The pituitaries of male SD rats aged 0, 2, 3, 4, 6, and 8 weeks were collected. Each age group contained three rats. The obtained tissues were stored in TRIzol reagent (Tiangen, Beijing, China) for subsequent experiments.

### 2.3. Cell Culture

All cell experiments in this study were performed on rat anterior adenohypophyseal cells that were cultured with Dulbecco’s modified Eagle’s medium/F12 (DMEM/F12, HyClone, Logan, UT, USA) containing 15% fetal bovine serum (FBS, Gibco, New York, NY, USA) and 1% penicillin/streptomycin. All rat anterior adenohypophyseal cells were cultured at 37 °C under a humidified atmosphere containing 5% CO_2_.

The pituitary tissues of eight-week-old male SD rats were collected for culturing rat anterior adenohypophyseal cells. Ten percent poly-1-lysine (Boster Biology, Pleasanton, CA, USA) was processed in advance in a six-well plate for use. After washing and removing the neurohypophysis, the adenohypophysis was transferred to DMEM/F12 containing 2.5% type I collagenase (Gibco, USA) and cut into pieces with ophthalmic scissors. Then, they were put into a water bath at 37 °C for 90 min, and the digested cell suspension was filtered through a 200 mesh (75 µm) sieve to separate the undigested tissues and cell aggregates and subjected to centrifugation at 200× *g* for 10 min. The obtained cell pellet was prepared in a cell suspension and seeded in a six-well plate prepared in advance. The details of the rat anterior adenohypophyseal cell culture procedure have been described in a previous study by our research group [17]. Male Simmental cattle aged 20–22 months were selected from the slaughterhouse, and pituitary tissues were collected for culturing bovine anterior adenohypophyseal cells. The same culture method was used to culture bovine anterior adenohypophyseal cells.

### 2.4. GnRH Analogue Treatment for Cows

**Figure 1:**
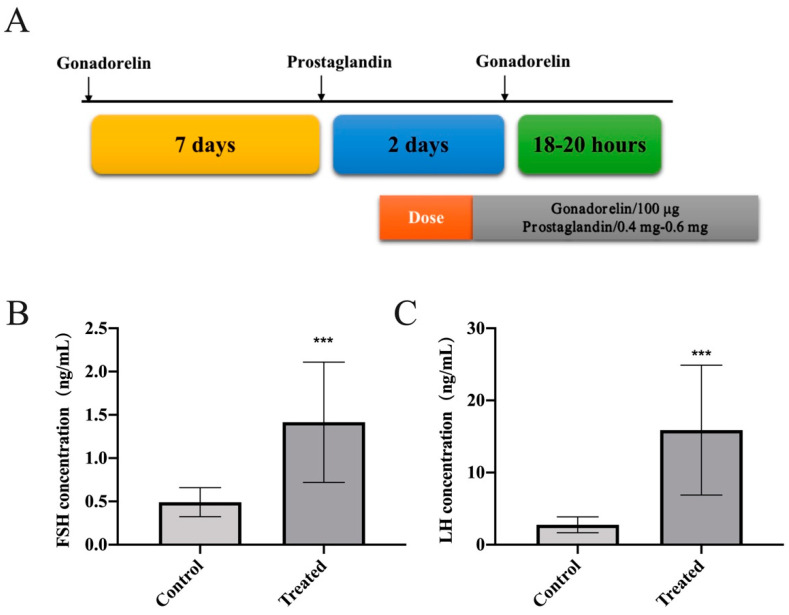
GnRH analogue treatment promoted the synthesis and secretion of FSH and LH in cows. (**A**) The combined treatment program diagram of gonadorelin and prostaglandin for cows. (**B**,**C**) ELISA was used to determine the FSH and LH secretion of cows after treatment. All experiments were repeated at least three times. Data are presented as the means ± SD. *p* < 0.05 was considered significant. When *p* < 0.001, *** was used to show the significance of the difference.

The cows were treated with a combination of gonadorelin and prostaglandin for estrus synchronization before insemination (Figure 1A). Gonadorelin was injected intramuscularly into each cow at a dose of 100 μg. After 7 days, each cow was injected with 0.4–0.6 mg of prostaglandin. After 48 h, each cow was again injected with 100 μg of gonadorelin. With this technique, insemination was able to be performed over approximately the next 20 h without observation of the estrus phase of the cows. Blood was collected from the jugular vein of each cow before the gonadorelin injection and 2 h after the second injection. The serum was stored at −20 °C for subsequent hormone detection after centrifugation.

### 2.5. GnRH Analogue Treatment for Rats

To prepare the GnRH injection solution, 100 ng of gonadorelin (GnRH analogue, Ningbo Sansheng Biotechnology Co., Ltd., Ningbo, China) was mixed uniformly in 10 mL of saline (Shanghai Baxter Medical Products Co., Ltd., Shanghai, China) to yield a 0.01 μg/μL mixture. Twenty microliters of the mixed solution were added to 980 μL of saline to create a GnRH injection solution containing 0.2 μg of GnRH analogue with a final volume of 1 mL.

Six 8 week old male SD rats were divided into a control group and a treatment group with three rats in each group. The rats were anaesthetized with a CO_2_ anesthesia machine, and blood was collected from the orbital vasculature. After blood collection, the rats were given intraperitoneal injections. The rats in the control group were intraperitoneally injected with 1 mL of saline, and those in the treatment group were intraperitoneally injected with 1 mL of GnRH. The same dose of saline or GnRH was injected again two hours later. Ten to fifteen minutes after the second injection, the rats were euthanized using the CO_2_ anesthesia machine, and 200 μL of blood and pituitary tissues were collected.

### 2.6. GnRH Treatment for Cells

The primary pituitary cells were digested with trypsin EDTA solution A (0.25% trypsin and 0.02% EDTA in Puck’s saline A) and were collected by centrifugation at 200× *g* for 5 min. Then, the cells were subsequently resuspended in 10% FBS supplemented DMEM and seeded at a density of 1.0 × 10^6^ cells/well in a 12-well plate prepared in advance. After incubation for 24 h in 5% CO_2_ humidified 37 °C air, the dispersed pituitary cells were treated with 100 nM GnRH-I (MCE, China) for up to 8 h in serum-free DMEM/F12, followed by RNA extraction as described below.

### 2.7. Transfection

Rat anterior adenohypophyseal cells were cultured in a 24-well plate at a density of 3 × 10^5^ cells per well. A Lipofectamine 2000 Transfection Kit (Thermo Fisher Scientific, Waltham, MA, USA) was used for transfection according to the manufacturer’s protocol. The final concentrations of miRNA mimics, inhibitors, and negative controls were all 100 nM. After transfection, the cells were incubated in a 5% CO_2_ incubator for 24 h to provide adequate time for the reaction to proceed. All mimics, inhibitors, and siRNAs were purchased from RiboBio (Guangzhou, China), and the transfection methods and processing steps were performed in strict accordance with the manufacturer’s recommended protocols.

### 2.8. RNA Extraction, Reverse Transcription, and Real-Time q-PCR

Rat anterior adenohypophyseal cells were treated with TRIzol reagent (Tiangen, Beijing, China) after transfection, and RNA was extracted from the collected tissues and cells. A NanoDrop ND-2000 spectrophotometer (NanoDrop Technologies, Beijing, China) was used to measure the concentration and purity of RNA to verify that the protocol and RNA quality met the standards. A FastQuant RT Kit (with gDNase, Tiangen, China) was used for reverse transcription to obtain cDNA, and SuperReal PreMix Plus (SYBR Green, Tiangen, China) was used to detect the mRNA expression of related genes by real-time q-PCR according to the manufacturer’s recommended protocol. All primers used in PCR and real-time q-PCR are shown in Table 1.

### 2.9. Dual Luciferase Reporter Assay

**Figure 2:**
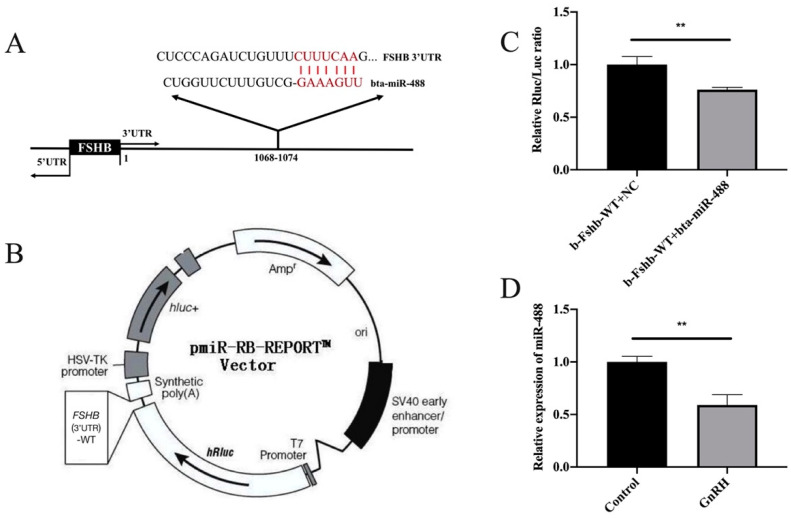
MiR-488 targeted the *FSHB* 3’UTR. (**A**) The complementary base pairing region of miR-488 and *FSHB* predicted through TargetScan program is shown in red. (**B**) The vector map of pmiR-RB-REPORT^TM^ Vector. (**C**) The relative luciferase activity was measured after the co-transfection of pmiR-Fshβ-3’UTR-WT with the miR-488 negative control and mimic. (**D**) The expression of miR-488 after GnRH analogue treatment in bovine anterior adenohypophyseal cells. All experiments were repeated at least three times. Data are presented as the means ± SD. *p* < 0.05 was considered significant. When *p* < 0.01, ** was used to show the significance of the difference.

The pmiR-Fshβ-3’UTR-WT plasmid was constructed with the assistance of RiboBio Biotech Co., Ltd. (Figure 2B). All construct products were confirmed by sequencing (RiboBio Biotech Co., Ltd., Guangzhou, China). For the analysis of the targeted binding relationship between miR-488 and Fshβ mRNA, 293T cells were co-transfected with miR-488 mimic, negative control, pmiR-Fshβ-3’UTR-WT, and pmiR-RB-REPORTTM. The luciferase activity was measured using a fluorescence intensity meter (Veritas 9100-002) after transfection for 48 h. Renilla luciferase was used as an internal reference luciferase to minimize experimental variability. All these experiments were repeated at least three times.

### 2.10. ELISA

Blood samples were collected from eight-week-old male SD rats before intraperitoneal injection (negative control group, three replicates) and 2 h after intraperitoneal injection of the GnRH analogue (experimental group, three replicates). The blood was centrifuged at 12,000× *g* for 10 min, and the supernatant was collected. A rat FSH ELISA kit (Meilian Biotech Co., Ltd., Shanghai, China) and a rat LH ELISA kit (Meilian Biotech Co., Ltd., Shanghai, China) were used to detect changes in the FSH and LH levels in the blood of the rats before and after GnRH analogue treatment.

For cow blood samples detection, the blood was centrifuged at 2000 rpm/min for 20 min, and the supernatant was collected. The corresponding kit needed to be replaced. A bovine FSH ELISA kit (Meilian Biotech Co., Ltd., Shanghai, China) and a bovine LH ELISA kit (Meilian Biotech Co., Ltd., Shanghai, China) were used to detect changes in the FSH and LH levels in the blood of the cows, and the rest of the steps were completed according to the kit’s instructions.

After transfection for 24 h, the DMEM/F12 (containing 15% FBS) in which rat anterior adenohypophyseal cells were cultured was replaced with serum-free medium. After culturing for another 24 h, 50 μL of the cell supernatant was collected to detect the changes in FSH levels using a rat FSH ELISA kit (Meilian Biotech Co., Ltd., Shanghai, China).

### 2.11. Apoptosis Analysis

Flow cytometry was used to detect the apoptosis of rat anterior adenohypophyseal cells in order to evaluate the effect of transfection after 24 h. Cells were digested with trypsin EDTA solution A (0.25% trypsin and 0.02% EDTA in Puck’s saline A) and transferred to a 10 mL reaction tube at an appropriate time point after transfection. The cells obtained in the previous step were collected by centrifugation at 200× *g* for 5 min and resuspended in 500 μL of 1× working fluid (5× binding buffer diluted with double-distilled water to 1× working fluid). Then, 5 μL of Annexin V-fluorescein isothiocyanate (FITC) and 10 μL of propidium iodide (PI) were added to a blank control tube and to the sample tube containing the above cell suspension with an Annexin V-FITC/PI Apoptosis Kit (Multi Sciences, Hangzhou, China) according to the manufacturer’s instructions. Apoptosis was analyzed by flow cytometry for 1 h.

### 2.12. MiRNA Target Prediction

In order to verify whether miR-488 can target the 3’UTR of the *FSHB*, we used the TargetScan program (http://www.Targetscan.org/) (accessed on 21 April 2020) to predict the miRNAs that might target the 3’UTR of the *FSHB* mRNA. Entering the sequence of the *FSHB* 3’UTR into the program can predict miRNAs that may have a targeting relationship with *FSHB*.

### 2.13. Statistical Analysis

All data are presented as the means ± standard deviations from three independent experiments. Multiple comparisons of data were performed with one-way ANOVA using SPSS 19.0 for Windows to obtain the significant differences. *p* < 0.05 was considered to indicate statistical significance.

## 3. Results

### 3.1. GnRH Analogue Treatment Promoted the Synthesis and Secretion of FSH and LH in Cows

GnRH analogues, which can affect the secretion of reproductive hormones and promote ovulation, can be used for estrus synchronization. To explore the effects of GnRH analogue treatment on FSH and LH secretion in cows, gonadorelin and prostaglandin were combined to achieve estrus synchronization. Cow serum was collected, and the changes in the two gonadotropins were detected by ELISA. The results showed that the secretion of FSH and LH was significantly upregulated after GnRH analogue treatment (Figure 1B,C), indicating that GnRH analogues can promote the synthesis and secretion of FSH and LH.

### 3.2. MiR-488 Targeted the FSHB 3’UTR

To further explore whether a certain non-coding RNA participated in the process by which the GnRH analogue regulated FSH synthesis and secretion, miRNAs that target *FSHB* mRNA were predicted with the TargetScan prediction website (http://www.Targetscan.org/) (accessed on 21 April 2020). Among them, miR-488 was selected because of its highly conserved sequence (Figure 2A). A dual luciferase reporter assay was used to further verify that miR-488 targeted *FSHB*. As expected, the luciferase activity was reduced (by 43%) after co-transfection (Figure 2C). In addition, we found that the expression of miR-488 was significantly downregulated after GnRH treatment in bovine anterior adenohypophyseal cells (Figure 2D). Therefore, we speculate that miR-488 may regulate *FSHB* expression by directly targeting *FSHB* mRNA.

### 3.3. Low-Frequency GnRH Analogue Treatment Promoted the Synthesis and Secretion of FSH and LH in Rats

**Figure 3:**
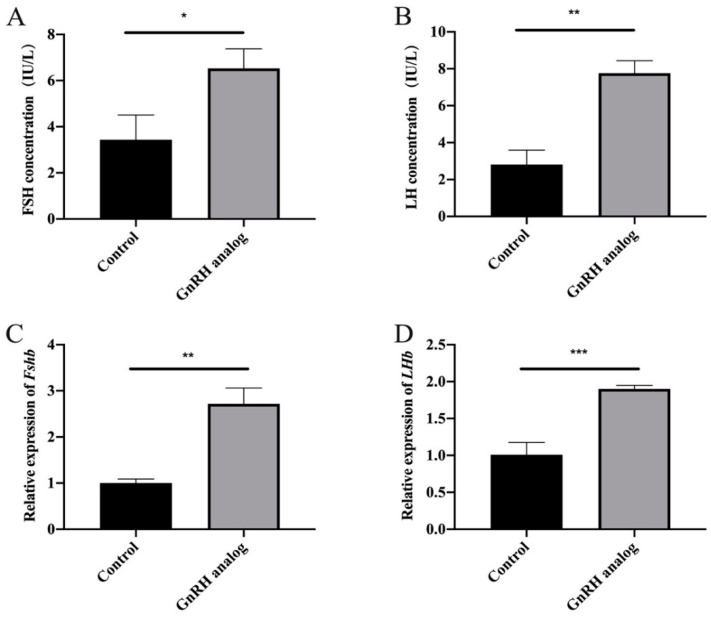
Low-frequency GnRH analogue treatment promoted the synthesis and secretion of FSH and LH in rats. (**A**,**B**) The secretion levels of FSH and LH after treatment with the GnRH analogue. (**C**,**D**) The expression of *Fshβ* mRNA and *Lhβ* mRNA after treatment with the GnRH analogue. All experiments were repeated at least three times. Data are presented as the means ± SD. *p* < 0.05 was considered significant, and the differences are marked with the symbol *. When *p* < 0.01, ** was used to show the significance of the difference. When *p* < 0.001, *** was used to show the significance of the difference.

Considering the difficulty of manipulating cattle and the identical sequence of miR-488 between cattle and rats (i.e., UUGAAAGGCUGUUUCUUGGUC), we used rats and rat anterior adenohypophyseal cells to explore the mechanism by which the miRNA regulated FSH synthesis. To explore the regulatory effect of pulsed GnRH on FSH, rats were first treated with the GnRH analogue at a low frequency. Serum was collected to detect FSH and LH secretion by ELISA, and the pituitaries of the rats were collected to detect the expression of *Fshβ* mRNA and *Lhβ* mRNA by RT-qPCR. The secretion levels of FSH and LH increased significantly after treatment with the GnRH analogue (Figure 3A,B). Additionally, the expression of *Fshβ* mRNA and *Lhβ* mRNA were significantly upregulated (Figure 3C,D). These results indicate that low-frequency GnRH analogue treatment can promote the synthesis and secretion of FSH and LH in rats.

### 3.4. Regulation of FSH Synthesis by Differentially Expressed miR-488 in Rat Anterior Adenohypophyseal Cells

**Figure 4:**
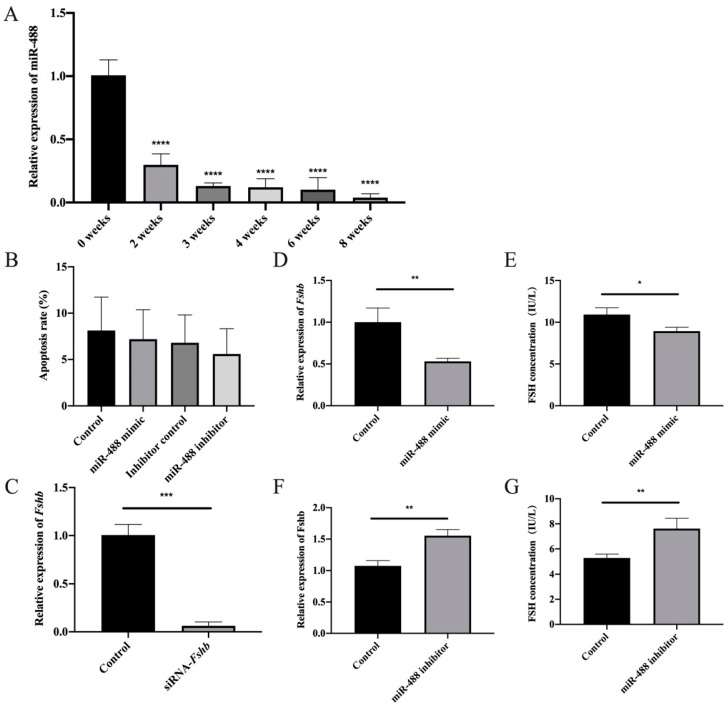
Regulation of FSH synthesis by differentially expressed miR-488 in rat anterior adenohypophyseal cells. (**A**) The expression of miR-488 in the pituitary glands of rats aged 0, 2, 3, 4, 6, and 8 weeks. (**B**) The apoptosis rate of rat anterior adenohypophyseal cells after transfection. (**C**) The relative expression of *Fshβ* after transfection with siRNA. (**D**–**G**) The levels of *Fshβ* mRNA expression and FSH synthesis after the transfection of a miR-488 negative control sequence, mimic, and inhibitor into rat anterior adenohypophyseal cells for 24 h. All experiments were repeated at least three times. Data are presented as the means ± SD. *p* < 0.05 was considered significant, and the differences are marked with the symbol *. When *p* < 0.01, ** was used to show the significance of the difference. When *p* < 0.001, *** was used to show the significance of the difference. When *p* < 0.0001, **** was used to show the significance of the difference.

The expression of miR-488 at various developmental stages in the adenohypophysis was analyzed to gain insight into miR-488 expression patterns. Specifically, RT-qPCR was used to detect the expression of miR-488 in the pituitary glands of rats aged 0, 2, 3, 4, 6, and 8 weeks. The expression of miR-488 was significantly downregulated in the pituitary glands of sexually mature rats compared to those of immature rats (Figure 4A). This result is consistent with the physiological phenomenon that FSH synthesis in rats is greater after sexual maturity is reached than during the immature period.

To further confirm the regulatory effect of miR-488 on FSH, transfection experiments were performed on rat anterior adenohypophyseal cells. Briefly, the effects of knockdown or overexpression of miR-488 on *Fshβ* mRNA expression and FSH synthesis were determined. Flow cytometry was used to detect apoptosis in order to assess the cytotoxicity of the transfection reagent. No significant difference in apoptosis was observed among the control group, mimic group, and inhibitor group, indicating that the effect of the transfection reagent on the cells was negligible (Figure 4B).

miR-488 mimics and inhibitors were transfected into rat anterior adenohypophyseal cells, and then the levels of *Fshβ* mRNA expression and FSH synthesis were tested to further confirm that miR-488 affected the expression of *Fshβ* and regulated animal reproduction. As a positive control, *Fshβ* siRNA was transfected for 24 h, and the expression level of *Fshβ* mRNA was detected by RT-qPCR. As expected, the expression level of *Fshβ* mRNA was significantly downregulated by siRNA transfection (Figure 4C). After a miR-488 negative control (NC) sequence and mimic were transfected into rat anterior adenohypophyseal cells for 24 h, the levels of *Fshβ* mRNA expression and FSH synthesis were also significantly downregulated by miR-488 mimic (Figure 4D,E). In contrast, the levels of *Fshβ* mRNA expression and FSH synthesis increased significantly after transfection with miR-488 inhibitors (Figure 4F,G). We also detected changes in FSHB protein levels after transfection. The experimental results were consistent with the above results. The original western blot figures are provided in the Supplementary Materials These results indicate that miR-488 has regulatory effects on *Fshβ* expression and FSH synthesis.

## 4. Discussion

GnRH, a key regulator of the reproductive system located at the top of the HPG axis, is the main regulator of FSH synthesis and secretion. Gonadorelin for injection is a type of synthetic GnRH that has become a commercial GnRH analogue for veterinary use [18]. Gonadorelin can regulate animal reproductive function by promoting the synthesis and secretion of gonadotropins in the adenohypophysis [19]. In addition, gonadorelin has therapeutic efficacy against HCG or LH secretion disorders, follicular ovulation disorders, and other diseases [20]. Gonadorelin is commonly used clinically to promote the recovery of ovarian function [21]. In addition, the combined use of gonadorelin with other treatments can significantly increase the pregnancy rates of cattle during estrus in the process of artificial insemination [22]. Therefore, exploring the mechanism by which GnRH regulates FSH secretion can provide a theoretical basis for improving livestock estrus control procedures and promote the domestic application of hormone products.

Based on this practical problem, we started from the perspective of simultaneous estrus processing in cattle and aimed to find a new molecular mechanism to construct a cattle model by injecting GnRH in vitro. As expected, our results showed that the secretion of FSH and LH for 30 cows was significantly upregulated after GnRH analogue treatment. However, the experimental data show that there are great differences between individuals, which is consistent with the actual situation that the efficiency of the same period of estrus has fallen into a bottleneck in the current production practice. We believe that this may be because the different individuals have different sensitivities to hormones, or because the feedback regulation of reproductive hormones is very complicated. It is believed that this phenomenon implies that there are still many unknowns regarding the molecular mechanism of GnRH regulating FSH.

Many studies have reported the involvement of miRNAs, the earliest discovered and most thoroughly researched type of non-coding RNA, in physiological processes in animals. MiRNAs play certain regulatory roles in transcription, translational repression, and gene expression [23,24,25]. In addition to basic biological processes, miRNAs are also involved in the formation of various organs in animals and apparently participate in regulatory and metabolic processes [26,27,28]. Notably, abnormal expression of miRNAs has been confirmed to be associated with the occurrence and development of various human diseases such as cancers [29], diabetes [30], cardiovascular complications [31], and neurodegenerative diseases [32,33]. In addition, miRNAs actively participate in the regulation of hormone secretion. Matarese et al. confirmed that miR-7 could regulate GLP-1-mediated insulin release by targeting β-arrestin 1 [34]. GH signaling is also affected by miRNA dysregulation [35]. Similarly, our team’s previous research revealed that miR-186-5p, miR-7a-5p, and other miRNAs are involved in FSH secretion regulation [36,37].

Therefore, through the target gene prediction software, we predicted that a variety of miRNAs might act on FSHB and discovered miR-488, which is highly conserved in the bovine anterior pituitary gland. By using exogenous GnRH to treat bovine anterior adenohypophyseal cells, the differential expression of miR-488 was successfully found. This implies that miR-488 may be involved in the regulation of FSH synthesis and secretion. Two recent studies have found that miR-488 could be used as a diagnostic biomarker of renal cell carcinoma and ovarian cancer [38,39]. Therefore, we hope to further explore the role of miR-488 in the regulation of reproductive hormone synthesis and secretion. Elucidating the mechanism of FSH secretion regulation will enable more potential functions of miRNA-488 to be unearthed.

The results of bioinformatics prediction and dual luciferase reporter assays in the current study indicated that miR-488 was able to target the *FSHB* mRNA 3’UTR. However, due to the difficulty of manipulating cattle and the identical sequence of miR-488 between cattle and rats, we chose rats and rat anterior adenohypophyseal cells for subsequent molecular mechanism verification.

It is generally believed that different GnRH pulse frequencies and amplitudes affect the FSH secretion mode by activating different pathways such as the cAMP signaling pathway and the MAPK signaling pathway [40]. Feedback regulation also makes the crosstalk among various reproductive hormones in the HPG axis more complicated [41]. At present, there are numerous relevant reports on the internal regulation mechanism of the HPG axis. Regarding FSH, Thompson et al. examined GnRH pulse frequency-dependent stimulation of *Fshβ* transcription mediated by activation of PKA and CREB as early as 2013 [42]. Therefore, we aimed to verify this molecular mechanism in rats and tried to simulate GnRH low-frequency stimulation through multiple gonadorelin treatments. The results showed that GnRH low-frequency stimulation significantly promoted the expression of *Fshβ* and *Lhβ* in the adenohypophysis of rats. The secretion of FSH and LH in rats also significantly increased. Moreover, the expression of miR-488 showed similar differences with cattle. Based on the understanding of the GnRH signaling pathway, we speculate that the expression changes of miR-488 after GnRH treatment may also be affected by one or several of the cAMP/PKA/CREB signaling pathway, PKC/MAPK signaling pathway, and Ca^2+^/CaMK II signaling pathway. There may even be unknown key factors or signaling pathways involved, but this requires us to dig and explore in future research.

MiR-488 is also highly conserved in rats. In order to further explore the potential functions of miR-488, we tested the expression changes of miR-488 in rat pituitary glands at different developmental stages. The results showed a trend of significant decline in expression as age increased. During the normal developmental process of male rats, the testes begin to decline 23–25 days after birth, enter the scrotum at 30–35 days old, and produce sperm in 45–60 days. With the arrival of reproductive development, puberty, and sexual maturity, the secretion level of gonadotropins will increase, which is basically consistent with the significant decrease in the expression of miR-488. However, due to the complexity of the animal body and the uncertainty of miR-488 function, we cannot fully use the secretion of FSH to explain such a significant decrease in miR-488 expression. This may also be closely related to other physiological processes in the body. When we understand miR-488 at a deeper level, we can better explain this phenomenon.

The results of in vitro experiments on rat anterior adenohypophyseal cells showed that exogenous GnRH treatment significantly downregulated the expression of miR-488 while upregulating the expression of *Fshβ*. MiR-488 inhibited FSH synthesis by targeting *Fshβ* mRNA. Based on the results of this study, we propose that GnRH can regulate the synthesis and synthesis of FSH through miR-488. We hope these findings will help clarify the molecular mechanism by which GnRH regulates FSH and support additional research on the regulatory functions of miRNAs in reproduction.

## 5. Conclusions

This study found that miR-488 in the pituitary gland could inhibit the expression of *FSHB* by targeting the 3’UTR of *FSHB* mRNA, thereby inhibiting the synthesis and secretion of FSH. We hope to provide a theoretical basis for the application of GnRH analogue in cattle artificial breeding, and a research foundation for improving the processing procedures of cattle estrus control and the domestic application of hormone products.

## Figures and Tables

**Table 1:** Primers used for RT-qPCR.

Species	Primer Name		Sequence (5’–3’)
Rat	*Gapdh*	Forward primer	GGAAACCCATCACCATCTTC
		Reverse primer	GTGGTTCACACCCATCACAA
	*Fshβ*	Forward primer	ATACCACTTGGTGTGAGGGC
		Reverse primer	TAGAGGGAGTCTGAGTGGCG
	*Lhβ*	Forward primer	CAAAAGCCAGGTCAGGGATA
		Reverse primer	GTACTCGAACCATGCTAGGACA
	*U6*	Forward primer	GCTTCGGCAGCACATATACTAAAAT
		Reverse primer	CGCTTCACGAATTTGCGTGTCAT
	*U6*	Reverse transcription	CGCTTCACGAATTTGCGTGTCAT
	miR-488	Reverse transcription	CTCAACTGGTGTCGTGGAGTCGGCAATTCAGTGAG
	miR-488	Forward primer	ACACTCCAGCTGGGGCCTTCCGGT
	universal reverse		CTCAAGTGTCGTGGAGTCGGCAA

## Data Availability

The data contained in the article have been submitted to the Support table.

## Supplementary Materials

The western blot figures of FSHB protein after transfection of miR-488 mimic and inhibitor are available online at https://www.mdpi.com/article/10.3390/ani11113262/s1.
