# Supplementary material for: Regulation of FSH Synthesis by Differentially Expressed miR-488 in Anterior Adenohypophyseal Cells
Source: Animals (Basel). 2021 Nov 15;11(11):3262. doi: 10.3390/ani11113262 (PMC8614264; doi:10.3390/ani11113262)
Supplement: Supplementary file 1 [file animals-11-03262-s001.zip › animals-1417347 - supplementary.pdf]

animals

Supplementary Materials:

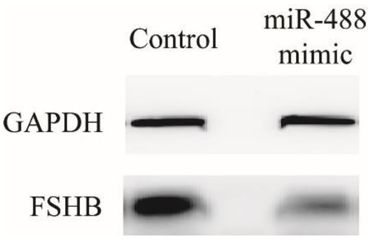

Figure S1. FSHB protein expression after transfection with miR-488 mimic.

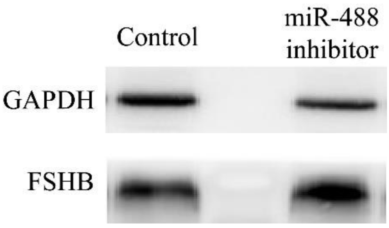

Figure S2. FSHB protein expression after transfection with miR-488 inhibitor.

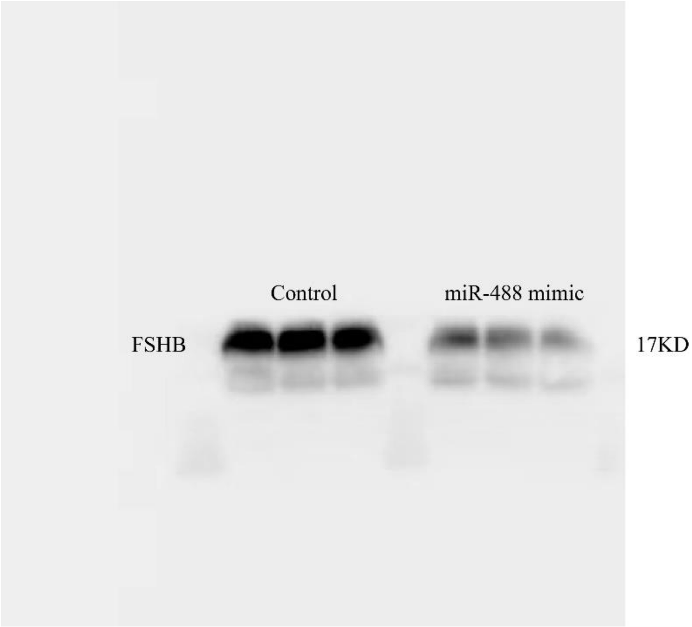

Figure S3. Original figure of FSHB protein after transfection with miR-488 mimic.

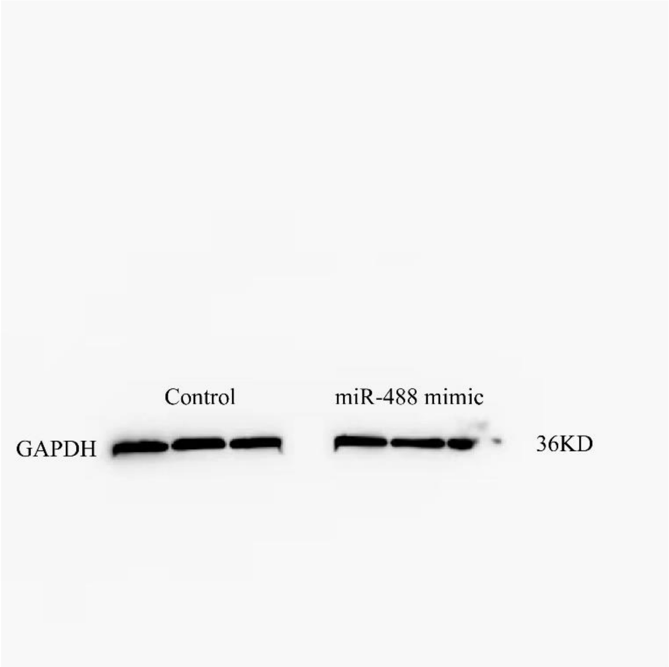

Figure S4. Original figure of GAPDH protein after transfection with miR-488 mimic.jpg.

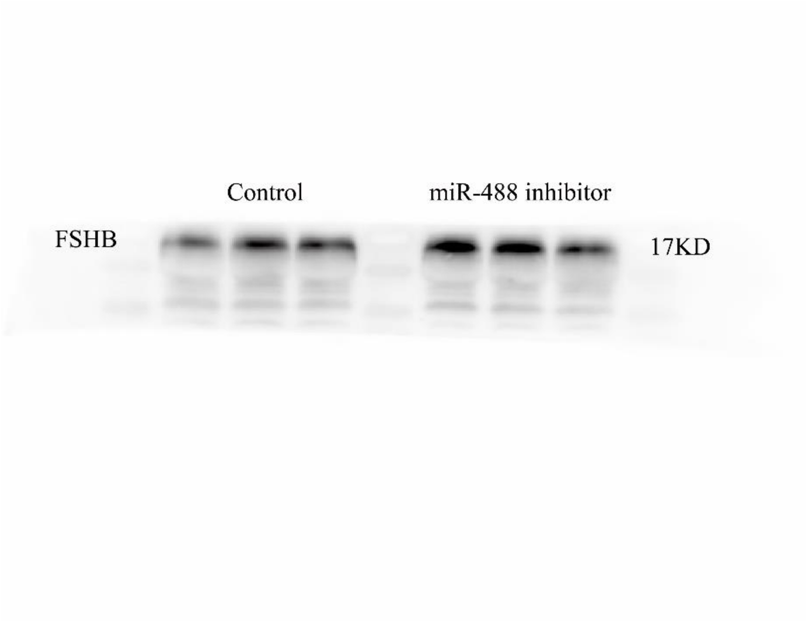

Figure S5. Original figure of FSHB protein after transfection with miR-488 inhibitor.

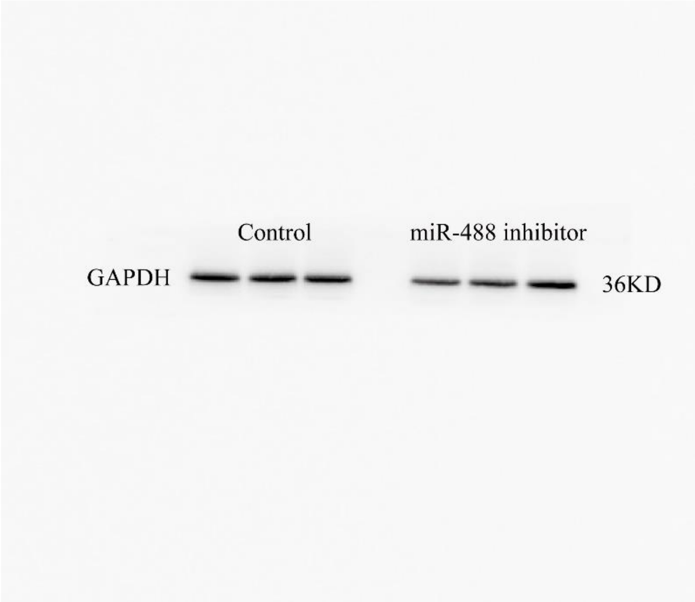

Figure S6. Original figure of GAPDH protein after transfection with miR-488 inhibitor.

Table S1. Raw data obtained from grayscale analysis.

	GAPDH	FSHB	FSHB/GAPDH
control	32523.945	14918.137	0.458681657
miR-488 mimic	27432.744	10786.472	0.393196977
	**GAPDH**	**FSHB**	**FSHB/GAPDH**
control	30087.116	28258.693	0.939229037
miR-488 inhibitor	29359.572	29806.288	1.015215344
